# A case of atlas assimilation: description of bony and soft structures

**DOI:** 10.1007/s00276-013-1235-9

**Published:** 2013-11-16

**Authors:** Maciej K. Ciołkowski, Paweł Krajewski, Bogdan Ciszek

**Affiliations:** 1 Department of Descriptive and Clinical Anatomy, Centre of Biostructure Research, Medical University of Warsaw, Chałubińskiego 5, 02-004 Warsaw, Poland; 2 Department of Forensic Medicine, Centre of Biostructure Research, Medical University of Warsaw, Warsaw, Poland; 3 Department of Neurosurgery, Children’s Memorial Hospital, Warsaw, Poland; 4 Department of Neurosurgery, Prof. Bogdanowicz Children Hospital, Warsaw, Poland

**Keywords:** Atlas assimilation, Vertebral artery, Suboccipital muscles, Suboccipital nerve

## Abstract

A case of atlas assimilation revealed during serial study of suboccipital region is presented. The specimen was harvested from the body of 31-year-old woman. Images of the computed tomography scans are correlated with classic dissection. Asymmetrical bony assimilation is accompanied by asymmetrical development of the suboccipital musculature. In the presented case, the atlantic segments of both vertebral arteries preserved their usual course between bony elements derived from the atlas and proatlas. Development of the soft tissues must be influenced by similar factors as development of the skeleton. Detailed radiologic studies, possibly with volumetric reconstructions, are necessary in cases of atlas assimilation before surgical interventions in the region of craniovertebral junction.

**Electronic supplementary material:**

The online version of this article (doi:10.1007/s00276-013-1235-9) contains supplementary material, which is available to authorized users.

## Introduction

Congenital fusion of atlas vertebra with occipital bone, known also as atlas assimilation, is anomaly observed in 0.08–2.76 % of population according to different authors [11]. While only sporadic osteological specimens were formerly presented by anatomists [2–4], development of diagnostic imaging allowed more systematic studies of larger patient series [6, 11]. Among patients with different craniovertebral abnormalities, there are 10.4 % of those with atlas assimilation [7]. These works revealed wide spectrum of morphological findings and more or less directly associated symptoms. Atlas assimilation may be asymptomatic or may cause noticeable limitation of neck flexion/extension movements with subsequent adjacent segment syndrome, or even form a base or element of serious vascular and neurologic problems, like vertebro-basilar insufficiency, atlanto-axial instability, and stenosis of the spinal canal at the level of craniovertebral junction or Chiari syndrome [5, 6, 9].

## Methodology and case report

During serial study of suboccipital region, a specimen with atypical morphology of the lateral parts of occipital bone was encountered. The block specimen containing posterior halves of the atlanto-occipital joints, posterior boundaries of the foramen magnum and posterior arch of atlas was harvested during routine forensic autopsy of 31-year-old woman, who suddenly died of pneumonia. The vertebral arteries were injected with coloured gelatine and the specimen was fixed in formalin. Measurements were taken with calliper. When atypical bony structure was noted the dissection was interrupted and a computed tomography (CT) scan was performed using CereTom™ NL3000 with slice thickness of 1.25 mm. Multiplanar and volume reconstructions were analysed on the working station of the CT machine and with OsiriX 5.6 **©**Pixmeo Sarl.

**Fig. 1:**
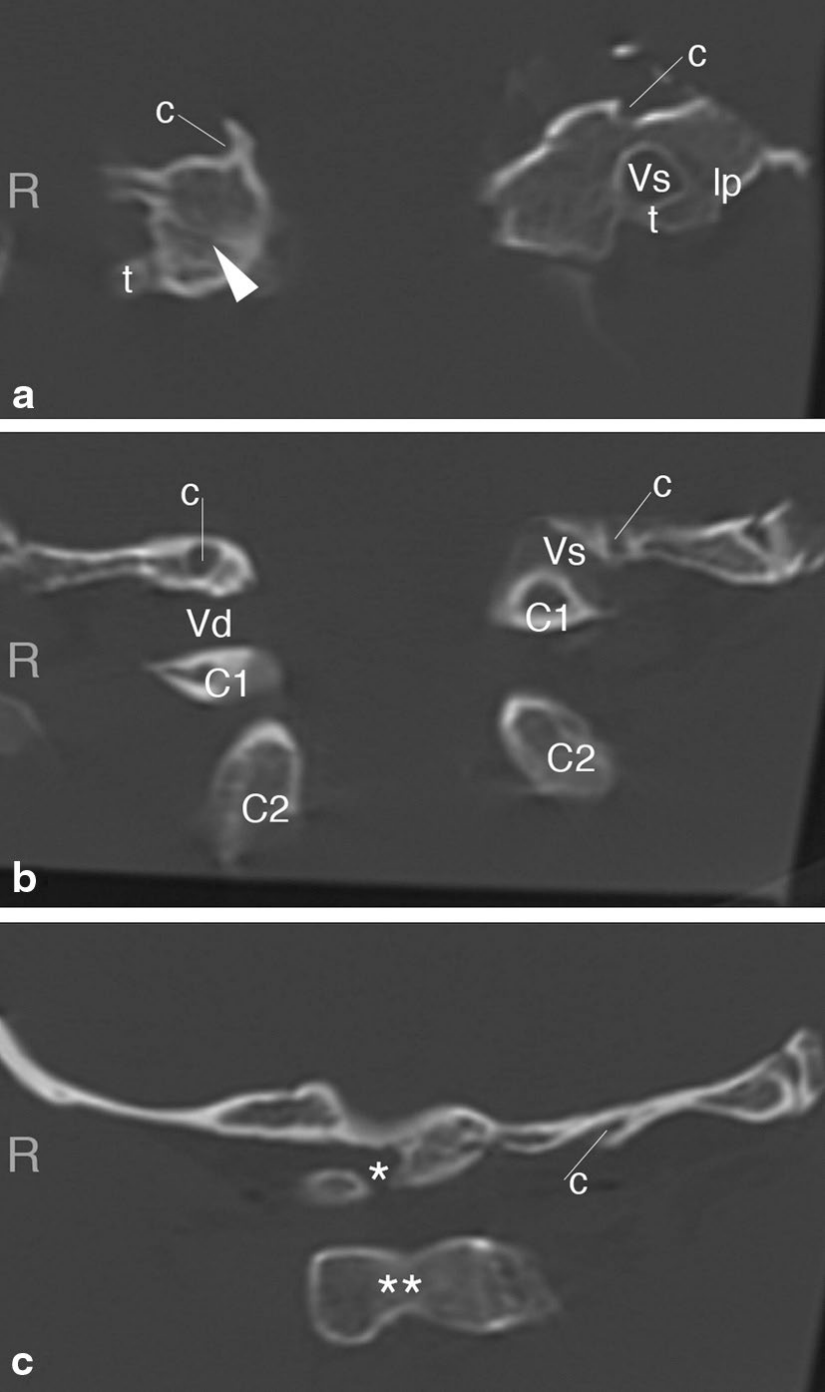
Coronal sections through the specimen (CT scan, bone window). **a** Most anterior, at the level of fused atlanto-occipital joints: *arrowhead* shows a line of fusion visible on the right but not on the left side, *t* posterior bar of the transverse process of atlas, *lp* lateral ponticle, *c* condylar emissary canal. **b** Middle, at the level of laminae of vertebral arches: *C1* posterior arch of atlas, *C2* lamina of arch of axis, *Vd* groove for the right vertebral artery, *Vs* canal for left vertebral artery shadowed by its posterior wall homologous with the posterior ponticle. **c** Most posterior, at the level of spinous processes: *dysraphic posterior arch of atlas, ** base of spinous process of axis

Preserved parts of both lateral masses of atlas were completely integrated with the occipital bone. Frontal CT and anatomical sections just anterior to the posterior arch pedicle revealed on the right side a plate of condensed trabecular bone at presumed level of atlanto-occipital joint, without any macroscopic vestigial cartilage. Despite this difference there was no significant asymmetry in height of the occipital condyle—lateral atlantic mass complexes (Fig. 1a). The posterior arch of atlas showed in the midline a dysraphic cleft 2.7 mm wide, filled with dense connective tissue (Fig. 1c). The right lamina was not connected to the occipital bone. The biggest distance between the right lamina and occipital bone was at the level of marked groove for vertebral artery (5.4 mm), and the smallest at the level of medial border of the groove (1.8 mm). The left lamina was smaller and fused with the margin of the foramen magnum, leaving only an ‘s’ shaped, narrow (4.4 mm) canal for the vertebral artery in its usual location (Fig. 1a, b). The condylar canals were present bilaterally above spaces for the vertebral arteries. The transverse processes were preserved on both sides. Additionally there was bony fusion between the tip of the left transverse process (at the level of its posterior bar) with paramastoid process of the occipital bone resembling the *ponticulus lateralis* of some atlas vertebrae. Total width of the assimilated atlas was 72.6 mm. Width of the foramen magnum was 30.9 mm, but at the level of atlas was 28.7 mm.

**Fig. 2:**
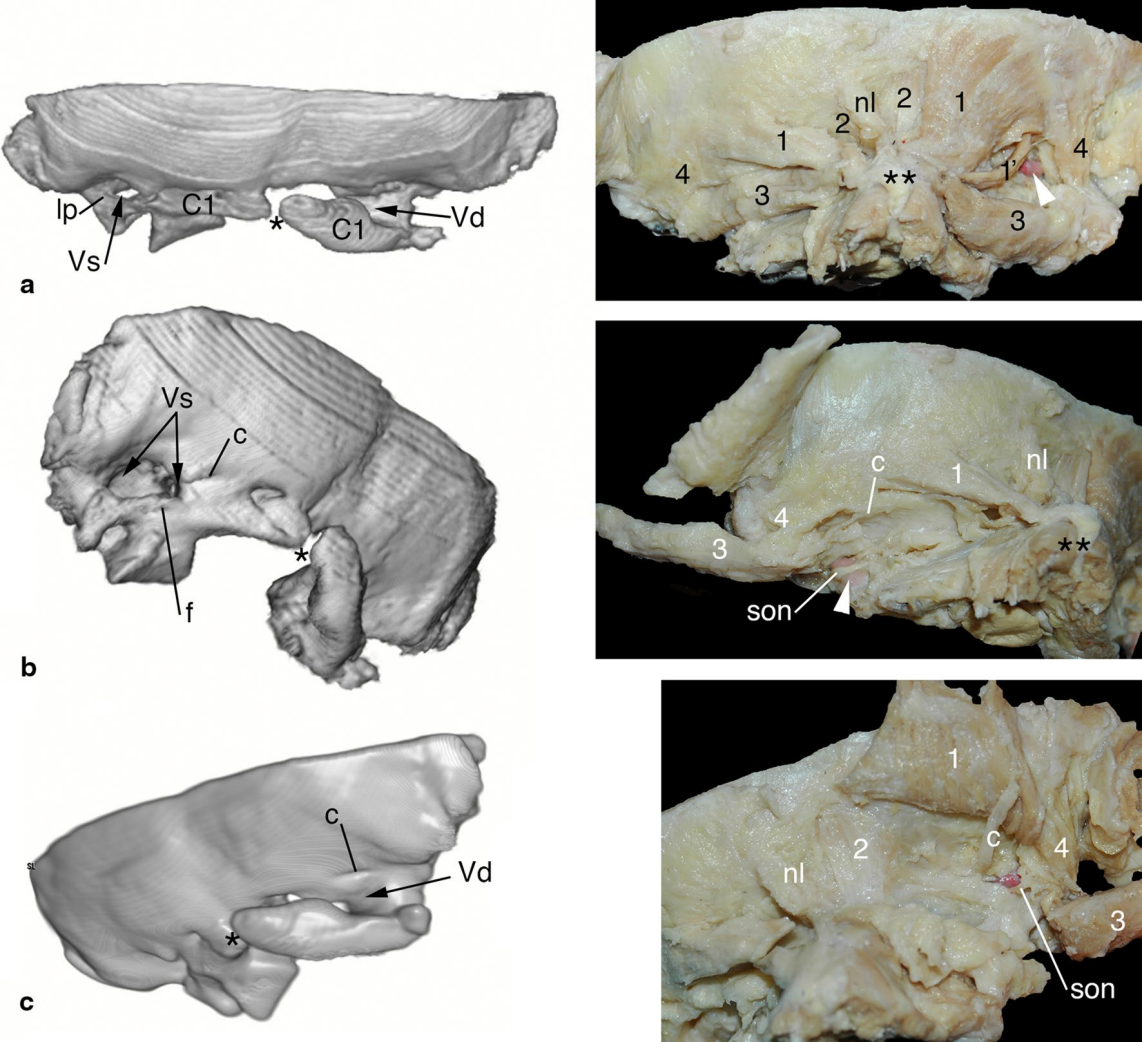
Three-dimensional reconstructions of the bony parts correlated with anatomical dissections. The arch of axis was removed from the reconstructions for clarity. In the posterior view (**a)** asymmetry of bony assimilation and muscular development is evident. The vertebral artery (*white arrowhead*) is visible after dissection of the suboccipital triangle on the right side, while on the left it is hidden in the bony canal and behind hypotrophic muscles and atlanto-occipital membrane: *C1* posterior arch of atlas, *lp* lateral ponticle, *Vd* groove for the right vertebral artery, *Vs* canal for left vertebral artery, *nl* nuchal ligament, *1* rectus capitis posterior major muscle, *1′* its accessory belly, *2* rectus capitis posterior minor, *3* obliquus capitis inferior, *4* obliquus capitis superior, *dysraphic posterior arch of atlas, **base of spinous process of axis. Posterior left oblique view (**b**) shows the bony canal containing left vertebral artery (*Vs*, *white arrowhead*) and surrounding structures: *c* aperture of the condylar emissary canal and its vein, *f* additional venous foramen, *son* suboccipital nerve. On the right oblique view (**c**) better-developed muscles on the right side are visible. Colour images can be viewed in electronic version and rotating 3D reconstructions are accessible as supplementary material

Suboccipital muscles preserved in the specimen were visually paler on the left side, which may indicate smaller contents of contractile fibres (Fig. 2). The obliquus capitis inferior muscle was developed bilaterally, the left being approximately a one-third thinner. The obliquus capitis superior on the right side was surprisingly well-developed, however, slightly discoloured compared to neighbouring muscles. On the left side, instead of the obliquus capitis superior there was a thick and short triangular bundle of connective tissue extending between the vestigial transverse process of the atlas and occipital squama. Like in normal situation it was directly posterior to the membrane (homologous with the posterior atlanto-occipital membrane) covering the vertebral artery, but distinguishable from it. The left rectus capitis posterior major muscle was much smaller than the right one, which consisted of two fan-like bellies. The right rectus capitis posterior minor was vestigial—short, thin, narrow, with long tendon attached to the free end of the right lamina of posterior atlantic arch. On the left side instead of the muscle there was a band of connective tissue, poorly distinguishable from the surrounding. The slit between the squama and preserved elements of the posterior arch was filled with multilayer fibrous connective tissue surrounding islets of fat and firmly attached to periosteum. The nuchal ligament was firmly attached to the external occipital crest, spinous process of the axis vertebra and fused with connective tissue filling the vestigial atlanto-occipital space and dysraphic slit of atlas.

The vertebral arteries were present bilaterally and had usual course. The right vertebral artery left the transversary foramen, arched posteriorly around the complex of lateral mass and occipital condyle and finally pierced the dura mater. Diameter of the right artery measured at the most posterior point of the atlantic part was 3.5 mm, and just behind the dura mater it was 3.1 mm. The left artery was running within partially open bony canal: first above the transversary foramen, medial to the bony bridge in location of the lateral atlanto-occipital ligament, then posterior and medial to the fused atlanto-occipital joint, above the posterior arch otherwise completely unified with the occipital squama on this side. A ‘window’ within osseous canal allowed the artery to form significant arch projecting posteriorly and inferiorly (Fig. 2b). Diameter of the left vertebral artery just below the transversary foramen was 3.0 mm, and just behind the dura mater it was 2.8 mm. Distance between the vertebral arteries piercing the dura mater was 24.4 mm.

On the medial aspect of the right vertebral artery the posterior spinal artery branched off extradurally. Its diameter was 1.0 mm. In the subarachnoid space, this artery gave off a small recurrent dural branch. No other arterial branches were visualised. Large condylar emissary veins after leaving their canals anastomosed with veins surrounding the vertebral arteries and finally emerged from the suboccipital triangles.

The first spinal nerve accompanied the atlantic segment of the vertebral arteries on both sides. The right one had ventral and dorsal roots, while the left only motor. Both suboccipital nerves traversed under the vertebral arteries and they broke up into several muscular branches.

## Discussion

This is the first to our knowledge presentation of soft tissues dissection in a case of atlas assimilation, where parallel radiologic study was performed. Bodon et al. [1] have recently presented results of dissection of a specimen with atlas assimilation, in which bony and muscular asymmetry was similar to our specimen, but their specimen had been finally macerated.

Asymmetry of congenital atlanto-occipital fusion visible also in our case is frequently observed [6], with extreme form of advanced assimilation limited to a half-atlas described by Green [3]. This may be one of the factors compromising balance of the cervical spine and leading to faster development of spine degeneration, which further results in irritation and compression of nervous structures at different levels in cases of C1 assimilation. Specific overload of the median atlanto-axial joint in these cases may lead to development of ‘periodontoid pseudotumor’—collection of granulomatous tissue and hyperplastic cartilage under the tectorial membrane finally compressing the medulla [5].

Described vestigial suboccipital musculature suggests its genetical connection with proper C_0_–C_1_–C_2_ segments and not with functional units of cranio-cervical junction. The suboccipital muscles during their development must be influenced by the same or similar factors as the bony elements, which result in similar asymmetry of segmentation and hypoplasia. This may be explained by difference in concentration gradients of regulatory proteins on the left and right side [8].

Bony structure of canals present due to atlas fusion with occipital bone, which resembles different bony bridges observed in normal atlases (*ponticulus posterior*, *ponticulus lateralis*), supports theory of their origin from proatlas [8, 10].

The vertebral arteries described in our specimen run between elements of atlas and occiput despite their fusion, so they belong to the type III of Wang et al. [11], the most frequent one (65 %) in their radiologic material. This position of the arteries makes their surgical dissection and mobilisation extremely difficult. Existence of spinal branches of the atlantic segment of vertebral artery should be taken under consideration in cases of atlanto-occipital fusion.

## Conclusion

Despite only partial preservation of craniovertebral junction structures in the presented specimen, it gave a rare opportunity to study appearance of soft tissues in a case of condition known mainly from osteological investigations. Implementation of the CT technique allowed for analysis of bony morphology in the context of more common atlas vertebra variations. Multiplanar reconstructions of thin slice CT study are optimal for planning any surgical or vascular interventions in cases of atlas assimilation.

## Electronic supplementary material

Below is the link to the electronic supplementary material.

Supplementary material 1 (MOV 2789 kb)

Supplementary material 2 (MOV 2447 kb)

Supplementary material 3 (MOV 2657 kb)
